# Jejunal multiple perforations for combined abdominal typhoid fever and miliary peritoneal tuberculosis

**DOI:** 10.11604/pamj.2019.33.51.14664

**Published:** 2019-05-23

**Authors:** Michele Grieco, Giorgia Polti, Lara Lambiase, Diletta Cassini

**Affiliations:** 1 General Surgery Department, S Eugenio Hospital, Piazzale dell’Umanesimo 10, 00144 Rome, Rome, Italy; 2 Immunoinfectivology Department, Bambino Gesù Pediatric Hospital, Piazza di Sant’Onofrio 4, 00165 Rome, Rome, Italy; 3 Infectious Disease Department, Aurelia Hospital, Via Aurelia 860, 00165 Rome, Rome, Italy; 4 General Surgery, Policlinico Abano Terme, Piazza C Colombo 1, 35031 Abano Terme (PD), Padua, Italy

**Keywords:** Typhoid fever, typhoid intestinal perforation, tuberculosis, abdominal tuberculosis

## Abstract

Typhoid fever and tuberculosis, considered rare diseases in western countries, is still considered a notable problem of health issue in developing countries. The gastrointestinal manifestations of typhoid fever are the most common and the typhoid intestinal perforation (TIP) is considered the most dangerous complication. Abdominal localization of tuberculosis is the 6^th^ most frequent site for extra pulmonary involvement, it can involve any part of the digestive system, including peritoneum, causing miliary peritoneal tuberculosis (MPT). This is the case report of a 4 years old girl with multiple jejunal perforations in a setting of contemporary miliary peritoneal tuberculosis and typhoid fever occurred in “Hopital Saint Jean de Dieu” in Tanguietà, north of Benin. The patient was admitted in the emergency department with an acute abdomen and suspect of intestinal perforation, in very bad clinical conditions, underwent emergency laparotomy. The finding was a multiple perforations of the jejunum in a setting of combined abdominal typhoid fever and miliary peritoneal tuberculosis. Typhoid intestinal perforations and peritoneal tuberculosis are a very rare cause of non-traumatic peritonitis in western country, but still common in developing country. Considering the modern migratory flux and the diffusion of volunteer missions all around the world, the western surgeon should know this pathological entities, and the best treatments available, well known by surgeons with experience of working in developing countries. The combination of both TIP and MPT in the same patient, is a very rare finding which can worsen the outcome of the patient itself.

## Introduction

Typhoid fever and tuberculosis, were a major problem of public health in Europe during the 19^th^ century. Nowadays considered a rare disease in western countries, are a notable problem of health issue in developing countries. Typhoid fever is one of the main health challenge in impoverished, overcrowded areas of the developing countries related with the lack of safe drinking. It is generally transmitted by the faecal-oral route and is often endemic. The gastrointestinal manifestations are the most common and the typhoid intestinal perforation (TIP) is considered the most dangerous complications. The mortality is high with a rate fluctuating from 5% to 80% depending on the surgical and not surgical health assistance available [1]. Even if the incidence of typhoid fever have declined over the last decades, in 2010 the estimation of its occurrence was of 13.5 million of cases, the major of them located in sub-Saharan Africa and south-east Asia [2]. Tuberculosis is a chronic granulomatous disease, it is related with the infection on Mycobacterium tuberculosis. Well known since ancient Egypt and Greece, this disease was put under control in 1946 with antimicrobial therapy. Tuberculosis is transmitted from an infected person to a susceptible person in airborne particles, called droplet nuclei. In 2016, 10.4 million people fell ill with tuberculosis and 1.7 million died from the disease. The pulmonary localization is still the most common form, but any part of the body can be involved by the disease [3]. Abdominal tuberculosis, less common in western countries, constitutes a major problem in terms of public health in developing countries, associated with a high rate of morbidity and mortality [4]. It's the 6^th^ most frequent site for extra pulmonary involvement and it can involve any part of the digestive system, including peritoneum, causing miliary peritoneal tuberculosis (MPT). This is the case report of a young girl with multiple jejunal perforations, in a setting of contemporary miliary peritoneal tuberculosis and typhoid fever.

## Patient and observation

A 4 years old girl, was brought by the parents to the emergency unit of the “Hopital Saint Jean de Dieu” in Tanguietà, north of Benin at lunchtime. The parents referred abdominal pain and tenderness, fever, vomiting and anorexia since 48 hours, treated with local herbs infuse. The patient was agitated but confused, Glasgow Coma Scale 12 (E3, V4, M5), arterial blood pressure 75/50 mm Hg, heart rate of 135 beats/min, body temperature 38.9°C and respiratory rate of 22 cycles/min. Abdominal test revealed a remarkable symmetrical distention, with bulging flanks, positive fluid thrill. Crying on every attempt of abdominal palpation, which revealed tenderness and pain on all the abdomen. The percussion of the abdomen was dull on all the quadrants, no peristalsis. The patient was tachypnoic with no particular findings on the thorax examination. Abdominal ultrasound exam revealed diffuse and massive ascites of mixed liquid/solid content. Laboratory findings included a normal cell counts: white blood cells 1300 cells/mm^3^ (normal value 5000-10000 cells/mm^3^), hemoglobin 7.7 g/dl (normal value 12-15 g/dl), sodium 136 mEq/L (normal value 135-145 mEq/L), potassium 5.6 mEq/L (normal value 3.5-5.1 mEq/L), ESR 44 mm/h (normal value 3-13 mm/h). Obtained the consent from the parents, nasogastric and vescical catheters were positioned, and the patient was brought to the operatory room, candidate to emergency explorative laparotomy. Under general endotracheal anesthesia with halothane, the emergency laparotomy with midline incision, revealed 4 liters of purulent/alimentary free peritoneal fluid, massive retroperitoneal lymphomegaly, multiple caseous nodes diffuse on the peritoneum and the bowel, measuring about 0.5 to 4 mm of diameter, and some bigger nodules (up to 1.5 cm) covered by caseous mucus with the suspect aspect of miliary peritoneal tuberculosis. On the middle jejunum 3 major perforations on the antimesenteric side of the bowel were identified, surrounded by hyperemic and inflamated tissue with the typical aspect of typhoid bowel perforation.

**Figure 1:**
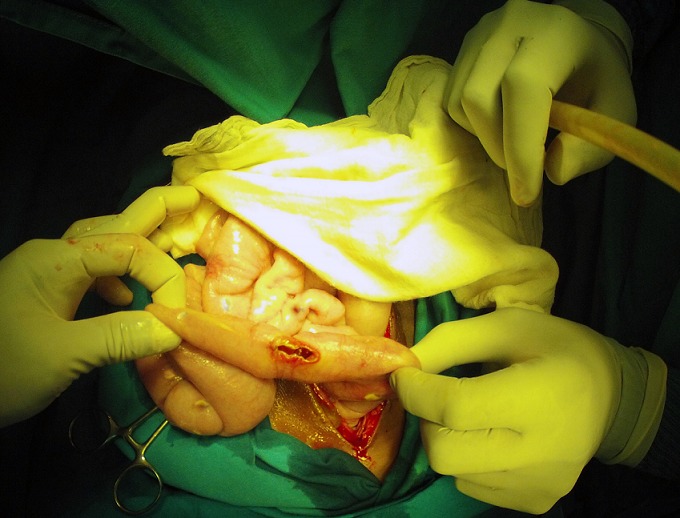
Surgical findings

**Figure 2:**
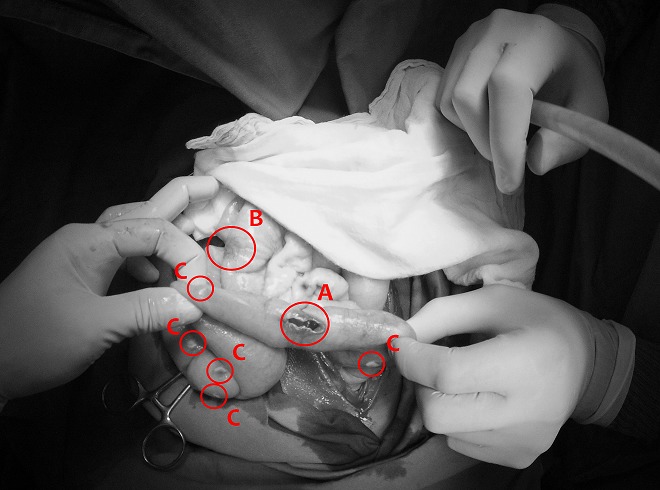
A) intestinal typhoid perforation; B) intestinal typhoid lesion not perforated; C) miliary peritoneal tuberculosis nodules

Some intestinal hyperemic intestinal typhoid lesions were also present, but not perforated yet. The surgical findings are shown in Figure 1, Figure 2. The surgical procedure included a lavage of the abdominal cavity and exploration of all the bowel for small lesion or perforations. A primary closure of the 3 perforations was performed with a separate stitch technique always used in the hospital [5]: a single-layer suture with 6 large vicryl 2/0 “U” stitches, through the seromuscular layer at 1cm from the perforation, to use the healthy tissue to close the perforation in a part of the bowel where there was less inflammatory involvement, with introflection of the perforation itself. A sample of the purulent material was taken for analysis, together with 2 peritoneal nodules. Considering the severity of the abdominal contamination, a delayed primary closure was planned, 3 drains were placed and a laparostomy was done fixing a grease gauze to the open aponeurosis with a continuous non-absorbable suture covered with sterile sponges. The samples sent for laboratory analysis showed a high load of acid fast bacilli on Ziehl-Neelsen staining and positive serology for Salmonella tiphy bacillus. The patient was very unstable throughout all the procedure, requiring correction of fluid and electrolyte imbalance, and one blood transfusion. A combined therapy of ciprofloxacine and metronidazole was administered intravenously ad the end of the surgical procedure for the treatment of the typhoid fever clearly recognized by the experienced local surgeon. A specifical treatment for the miliary peritoneal tuberculosis was not started due to the not certain origin of the peritoneal lesions. The general condition of the patient got worse hour by hour with a progressive septic shock and multi-organ failure. The young girl died 18 hours after the surgical procedure.

## Discussion

The “Hopital Saint Jean de Dieu” in Tanguietà, is located in the very north of Benin, in a rural area close to the border with Burkina Faso. It's a 300-bed facility serving a predominantly rural population coming even from Togo, Nigeria and Burkina Faso. It has basic operative facilities, but supportive care such as postoperative mechanical ventilation, parenteral nutrition and invasive monitoring are unavailable. Few western doctors are continuously working in the hospital, for short periods, as volunteers. TIP is a well known disease for the amount of patient admitted in this situation, with an average of 100 patients per year [5]. Tuberculosis is also a well known disease, but MPT is a rare findings in that area, occurring in few patients per year. In this case the patient was in very bad clinical conditions before the procedure and the surgery was considered the only life-saving procedure possible. Based on the opinion of the experienced local surgeons, all very skilled with TIP and MPT, the authors believe that the perforations were basically related with the typhoid fever, but the contemporary abdominal tuberculosis worsen the general condition of the patient, influencing the fatal outcome occurred. Typhoid fever globally has high mortality rate, without effective treatment it can reach 10-30%, reduced to 1-4% with appropriate therapy [6]. TIP constitute an extremely serious complication of the disease, especially for children, it can occur from 1% to 38% of the patients with typhoid fever [7]. Most of the patients with intestinal perforations, especially in rural areas, wait long time before hospitalization. This is a big problem in terms of managing unstable patients with anemia, fluid unbalance or sepsis especially in areas with inadequate health facilities [8]. The TIP are normally multiple, throughout all the small bowel. The bowel appear thin and inflamated around the perforation, which is often irregular, with white/yellow margin. This clinical features are always associated with a massive mesenteric limphadenopathy. This is what happened to the patient of this case report, brought to the hospital approximately 48 hours later the TIP gave the first symptoms, and the exploration of the abdomen revealed the typical presentation. There is uniform agreement that the surgical approach for TIP is the unique effective and life-saving. Actually the type of surgical technique remains not uniform, but seems to have limited influence on the outcomes [9]. Several surgical strategies have been proposed to treat TIP but real guidelines have never been produced. There are some reason for that heterogeneity in literature: most of these procedures are performed in developing countries where rigorous data collection is difficult, with high risk of bias and often without any risk-adjustment to grade the severity of the disease. Most of the studies report small and heterogeneous populations. From the available perspective studies, seems that the severity of the disease, the clinical condition of the patient, the virulence of the salmonella and the duration of disease before surgical treatment have more impact on the outcomes than the surgical technique itself [10, 11].

Surgical bowel resection seems more effective for multiple adjacent perforations while primary repair of the perforations seems the choice for a single perforation or multiple distant perforations. Small tract resections or wedge resections are the most uncommon treatment. Some studies compare the technique but the results are controversial [12]. On the other hand, a delayed closure of the abdomen is always recommended for heavily contaminated abdomen and unstable patients, but the optimal type of closure is not standardized [13]. MPT remains a world-wide problem, despite the discovery of the causative organism for more than a century ago. Among communicable diseases it is the second cause of death in developing countries, after HIV [14]. The peritoneal disease is rare and its clinical manifestations are vague, often can mimic many other abdominal disease, causing important delay in diagnosis [15]. Peritoneal involvement may occur due to the spread of the mycobacterium from mesenteric lymph nodes, or spread from intestinal lesions. In MPT the abdominal cavity is studded with multiple caseine granulomas, but often with a diffuse thick and hyperemic reaction. Strictures can be present from cicatricial healing of lesions [16, 17]. The characteristic caseine granulomas can also cause adhesions and produce caseine yellow mucus. Three types of peritoneal tuberculosis have been described: 1) ascitic type, with multiple nodules of 1-2mm and accumulation of peritoneal fluid. 2) Dry type, with scars, fibrotic tissue and adhesions. 3) Glandular type, which presents typical mesenteric lymphopaty and inconstant presence of ascites [18]. The patient in this case report had an ascitic type of MPT, with multiple caseine nodules, some adhesions but not fibrotic. The main treatment of peritoneal tuberculosis is similar to pulmonary tuberculosis with oral antibiotic combined therapy for a period of, at least, 6 months or more, depending on the case. Surgical intervention is required for patient developing intestinal perforation, obstruction or stricture. Rare clinical presentations are dysphagia, odynophagia, dyspepsia and gastric outlet obstruction due to gastroduodenal or esophageal tuberculosis. Since the clinical presentations of abdominal tuberculosis are very non-specific and vague, the diagnostic criteria's are limited, pathological diagnosis is not always easy but indispensable for the diagnosis of tuberculosis. Mycobacterium tuberculosis can be found in peritoneal ascites or in peritoneal nodules. When a peritoneal nodule is analyzed, the mycobacterium is often in the central part of the nodule, the peripheral part is cluster of immune cells including macrophages, epithelioid cells, multinucleated giant cells, Langerhans cells and lymphocytes [19].

## Conclusion

Typhoid intestinal perforations and peritoneal tuberculosis are a very rare cause of non-traumatic peritonitis in western country, but still common in developing country. Considering the modern migratory flux and the diffusion of volunteer missions all around the world, the western surgeon should know this pathological entities, and the best treatments available, well known by surgeons with experience of working in developing countries. The combination of both TIP and MPT in the same patient is a very rare finding which can worsen the outcome of the patient itself.

## Competing interests

The authors declare no competing interests.
